# Variations in ray flower numbers of Common Daisy (*Bellis perennis L.*) – the hidden cues of the Fibonacci order

**DOI:** 10.1371/journal.pone.0348529

**Published:** 2026-08-17

**Authors:** Carsten Neumann

**Affiliations:** Department 1 of Geodesy, Section 1.4 of Remote Sensing & Geoinformatics, GFZ Helmholtz Centre for Geosciences, Potsdam, Germany; Virginia Commonwealth University, UNITED STATES OF AMERICA

## Abstract

In this study, I provide empirical evidence on the number of ray flowers in *Bellis perennis L.* to evaluate the widely accepted assumption that floral architecture and other natural phenomena follow numerical sequences such as the Fibonacci order. The aim is to contribute to a broader understanding of the mathematical principles underlying the organization of natural systems, particularly the interplay between deterministic patterns and inherent stochasticity. Between April and May 2024, a total of n = 563 *Bellis perennis* individuals were sampled across 34 distinct populations in the Federal State of Brandenburg, northeastern Germany. The number of ray flowers per individual follows a well-defined probabilistic distribution that is best described by the discrete negative binomial type II model and the continuous inverse gamma probability density function. While most individuals exhibit ray flower counts between 34 and 56, a pronounced frequency peak occurs between 40 and 48, with a mode of 42 and a median of 46, both stable across spatial and temporal sampling conditions. Notably, there are two distinct local deviations identified by both theoretical distributions that occur at ray flower counts of 34 and 55, corresponding precisely to two values from the Fibonacci sequence. These deviations are neither pure statistical outliers nor indicators of multimodality; rather, they represent mathematically constructible features within a stochastic framework. To characterize this phenomenon, I introduce the concept of “improbable recurrency”, referring to deterministic structures that emerge from, and are only detectable within, inherent stochastic patterns of natural systems. Improbable recurrency exemplifies the co-constitutive relationship between stochasticity and determinism, forming a shared quantitative logic through which the human mind interprets biological complexity.

## Introduction

Are numbers an inherent property of reality or just a human construct? This old philosophical question is still widely debated, particularly in the natural sciences, where scientists try to find empirical evidence for mathematical concepts [1–3]. In fact, numbers themselves are interchangeable entities; however, their rule-based deduction and combination have often been shown to describe natural dynamics with remarkable precision, culminating in physical laws, process-based models, and the emergence of fundamental constants that appear universally stable in space and time [4]. Particularly in the living nature, simple numerical sequences seem to have a high explanatory power for, e.g., describing population growth, phyllotactic patterns, body morphologies, packing arrangements, life cycles, genetics etc. [5–8]. In other words, nature seems to be organized along certain numerical expressions that can be discovered by the human mind. On the other hand, complex natural systems can often only be modeled by stochastic processes, using probabilities to approximate natural phenomena [9–11]. This leads to the foundational issue of whether mathematical concepts are genuinely deterministic for nature, or whether stochasticity is inherent in nature [12–14].

For example, the species-specific floral architecture of Asteraceae plant family (also known as Compositae), encompassing patterns of plant growth, and the arrangement of leaves, seeds, and floral organs, is commonly described by modal morphologies, i.e., traits that vary among individuals but are typified by their most frequent expression, often corresponding to distinct Fibonacci numbers [15–18]. Interestingly, modal morphologies are mostly regarded as numbers neglecting the fact that they originate as more or less sharply defined maxima from a continuous distribution of all possible plant phenotypic responses. Since every single intraspecific floral trait could be confessed as an assemblage of individual phenotypic responses integrated over (co-)evolutionary temporal processes and along multiple environmental gradients, the resulting phenotypic information could also be described by uni- or polymodal probability density functions that in fact represent numerical continua rather than discrete numerical sequences. Consequently, the mathematical determinism tends to oversimplify a still largely unexplored complexity of intraspecific phenotypic diversity [19–21].

Although phenotypic variation is well documented across a wide range of organisms, environmental conditions, and biotic interactions [22], its epistemological interpretation through the mapping of individual probability distributions along spatiotemporally heterogeneous trait continua, is rarely addressed. One of the most diverse families of flowering plants, the Asteraceae, exemplifies this heterogeneity through pronounced variation in physiological and morphological traits, such as the number and arrangement of flowers and floral organs [23–25]. Asteraceae floral morphologies are therefore well suited to map the potential range, distribution, and variation of species-level phenotypic responses, and thus to identify the numerical foundations for next-generation phenotyping in relation to ecological and genomic predictive modeling, particularly to distinguish deterministic constraints from stochastic variation.

In the present study, I want to reveal the probability distribution of a floral trait, the ray flower number of common daisy (*Bellis perennis L.*, hereinafter referred to as *Bellis p.*) and show if/how modal morphologies are formed. On that basis, I want to test if ray flower compositions follow distinct numbers like the Fibonacci sequence, and if/how they can be derived from the respective probability density function. *Bellis p.* is a perennial herbaceous plant belonging to the Asteraceae family with a composed inflorescence that consist of a characteristic floral head (Capitulum) of yellow disc flowers in the center and the white ray flowers (also referred to as disc and ray florets) at the circular edge. In most standard floras, the number of *Bellis p.* ray flowers is either unspecifically defined as “numerous” [26] or clearly indicated as Fibonacci numbers 34, 55 or 89 without giving appropriate references [27] or not regarded at all. In recent studies, *Bellis p.* ray flower numbers are only vaguely defined between 20 and even > 100 [28] even though empirical evidence is broadly missing between as well as within populations. In fact, intraspecific variations have been investigated on the basis of several other individual traits such as of secondary metabolites [29], physiology and growth [30,31] and genomic structure [32, 33] whereas there is still a gap in defining the underlying distribution of response ranges to clearly derive modes as standard floral morphological numbers.

## Materials and methods

**Fig 1:**
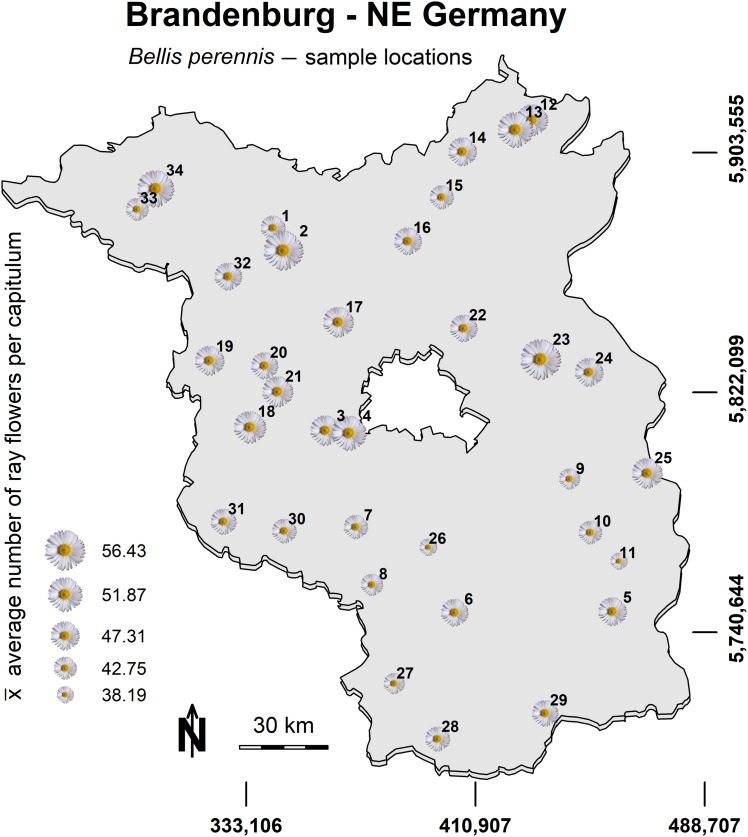
Spatial distribution of locations where *Bellis p.* populations were sampled; in each population between 10 and 20 specimens (individuals) were collected and ray flower numbers were counted; symbol size represents the average ray flower number per capitulum at each location, Brandenburg boundary source: © GeoBasis-DE/ BKG (2024), Verwaltungsgebiete 1:250 000 (VG250), dl-de/by-2-0.

The number of ray flowers was determined by counting the associated white petal strap (ligule), that is typically composed of 2 joined petals. The ligules were plucked out for counting. On average, every second batch was re-counted one to three times after plucking to assess counting consistency. Sample collection took place in spring between 3th April and 29th May in 2024 at n = 34 different locations in the Federal State of Brandenburg, North-East Germany (Fig 1). At each location, individual flower heads were sampled randomly from a local daisy flower population within an area of 50–1.000 m². Populations were detected during systematic surveys conducted from a moving car or by bicycle within and between settlement areas. Occurrences were recorded at playgrounds, backyards, cemeteries, village meadows, grass verges, and similar anthropogenic habitats. *Bellis p.* typically formed sharply defined population patches, whose natural boundaries clearly delimited the sampling area. Within each population patch, individuals were selected using a random point sampling procedure: a pencil was thrown n-times to generate random points within the bounded area, and the individual located closest to the pencil tip was collected. In average n = 17 specimens per population were collected, resulting in a total number of n = 563 specimens over all locations. While fine-scale spatial structure within patches was not explicitly quantified, the random point sampling procedure ensured that individual selection was unbiased by minimizing observer-driven choice with respect to spatial position. Only wild-type, single capitulum *Bellis p.* individuals with central yellow disc flowers were sampled; no obviously double-flowered morphs (e.g., horticultural garden escapes) were included. Temporal trend in individual ray flower counts were assessed using a univariate linear regression of all counts against collection time, with statistical significance evaluated based on the p-value of the regression coefficient. Spatial autocorrelation was additionally evaluated using Moran’s I, with neighbor relationships defined by a distance-based, row-standardized spatial weights matrix. Statistical significance was evaluated using permutation tests.

To characterize the frequency distribution of ray flower numbers, parametric count distributions were fitted using Generalized Additive Models for Location, Scale and Shape (GAMLSS) and maximum likelihood estimation [34,35]. Ray flower number was treated as a discrete response variable, and intercept-only models were specified to estimate population-level distributions across all sampled individuals. A set of discrete count distributions commonly employed for overdispersed ecological data was evaluated, including the Poisson, Negative Binomial (type II), zero-inflated Negative Binomial, and Delaporte distributions. Model selection was based on the Akaike Information Criterion (AIC; [36]), using the standard penalty (k = 2), to identify the distribution that best described ray flower numbers in *Bellis p.* across all individuals. In addition, a separate model selection procedure was applied to continuous models using the GAMLSS fitting routine for real-valued responses. These models were considered because ray flower numbers were relatively large and densely distributed, and because continuous probability density functions can provide insight into the stochastic structure of phenotypic variation, particularly with respect to tail behavior and variance scaling independent of integer constraints. Candidate distributions included continuous families defined on the real line (e.g., Normal, Logistic, Skew-Normal, Box–Cox–t, Johnson SU) as well as distributions restricted to the positive real line (e.g., Gamma, Lognormal, Inverse Gaussian, Inverse Gamma, Weibull, Generalized Gamma, and related Box–Cox transformations), as implemented in the GAMLSS family framework. Model parameters were estimated by maximum likelihood. Uncertainty was quantified using Wald-type standard errors and corresponding 95% confidence intervals derived from the observed information matrix on the link scale. Wald-type inference is appropriate in this context because GAMLSS parameters are estimated on transformed (typically logarithmic) scales, for which asymptotic normality of the estimators is assumed. Model fit was evaluated using worm plots, a de–trended quantile–quantile diagnostic that highlights deviations from the assumed distribution [37]. In the GAMLSS context, worm plots are based on normalized randomized quantile residuals [38] and facilitate assessment of variance, skewness, and tail behavior across the distribution [39]

Multimodality is shown by interpreting the threshold of bin sizes (frequency intervals) up to which distinct peaks can occur as fitted function maxima. Potential outlier counts were identified via a parametric bootstrap under the fitted theoretical distribution with 10,000 simulations. For each ray flower number, the 2.5% and 97.5% quantiles of the simulated counts were used to form a 95% confidence interval, and observed counts outside this range were flagged as potential outliers. For the continuous distribution model, expected counts were derived by integrating the fitted probability density over unit-width integer intervals, and simulated values were discretized to enable direct comparison with observed frequencies.

The best fitted probability density function was assumed to be the theoretical probability distribution for the continuous description of *Bellis p.* ray flower numbers. To delineate the occurrence of modal morphologies as true function maxima within the actual sample distribution of ray flower counts, a quantile-quantile comparison between the selected theoretical and the sample distribution was performed. Since a quantile-quantile comparison crucially depends on the quantile size, I performed a stepwise comparison of all quantile sizes between 2% and 20%. In each step, the difference between sample quantiles [SQ] and theoretical quantiles [TQ] are calculated and summed up over all steps [i] to retrieve the quantile residuals [RQ]. The residuals are finally plotted for all represented quantiles [Q] in the range of 29 ≤ Q ≥ 87 that gives:


$$\begin{array}{c} {RQ}_{n}=\sum_{i=\text{2}\%}^{\text{2}\text{0}\%}\left({SQ}_{i}-{TQ}_{i}\right)\;with\;n\;\in Q \end{array}$$ (1)

According to this, the final result reveals modal morphologies as positive or negative deviations of ray flower frequencies from their theoretical distribution function. While RQ allows to quantify the magnitude of deviation, n determines the width over which RQ is present which gives the metric RQ_n_ to finally evaluate the significance of function maxima.

## Results

Ray flowers were sampled for *Bellis p.* individuals from different populations distributed across the entire state of Brandenburg. The spatial distribution of *Bellis p.* ray flower numbers per capitulum averaged over all collected individuals at the respective location do not indicate spatial trends or autocorrelation within the study area (Fig 1). Close populations can have distinct differences in average ray flower counts, i.e., the maximum variance between populations is not spatially dependent on the considered spatial scale. Moran’s I was 0.072 and not statistically significant based on permutation testing (p = 0.237), clearly indicating a lack of evidence for spatial structuring in the response variable at the scale examined.

**Fig 2:**
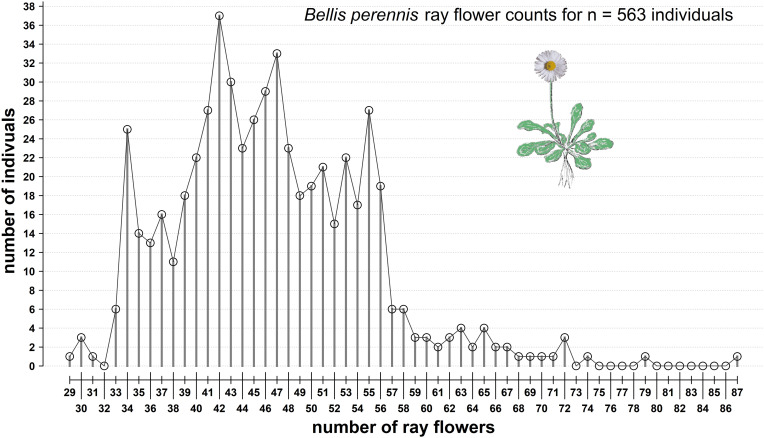
The number of *Bellis p.* individuals with specific ray flower counts for all n = 563 individuals in n = 34 different populations.

Most *Bellis p.* individuals do have ray flower numbers between 40 and 48 and 34 and 55 (Fig 2). Within the interval [40–48] high individual numbers were found, where two individual peaks at 42 and 47 are formed. Interestingly, the Fibonacci numbers 34 and 55 occur as sharp individual peaks. The whole range of all possible counted ray flowers is within 29 and 87. There are more extreme values at higher ray flower numbers; for example, the three maxima 74, 79 and 87 are counted only at one specimen, respectively, indicating a distribution skewed to the right. There is a second broader range detectable between 34 and 56 ray flower counts where most *Bellis p.* individuals fall in. Ray flower numbers lower and higher than this range are less frequent showing a steep drop in individual numbers. In conclusion, 3 separate modal morphologies of *Bellis p.* ray flower numbers can be distinguished; that is 1. the small range SR [40–48] with the highest number of individuals found, 2. the broad range BR [34–56] with most numbers of individuals found and 3. individual modes at M [34, 42, 47, 55] with sharply separated peaks of increased individual numbers found.

**Fig 3:**
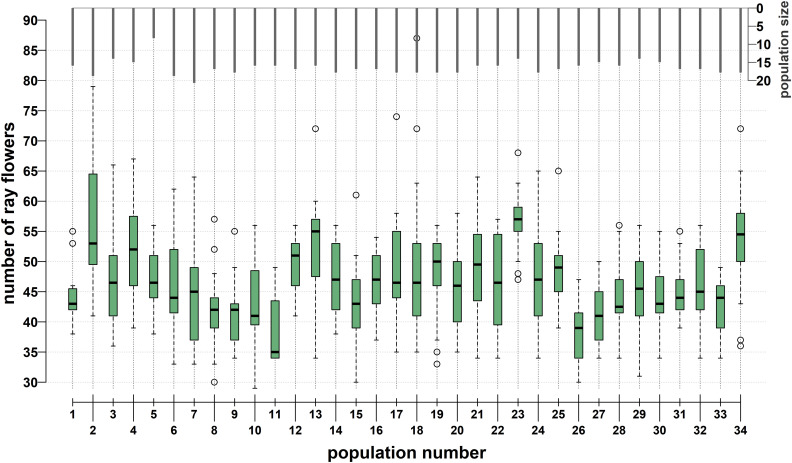
Box-and-whisker plots for all sampled locations (1–34), illustrating the distribution of ray flower numbers per individual within each population; population size corresponds to the number of individuals sampled at each location.

At the different sample locations, the within-population variation of ray flower numbers is high (Fig 3). While 88% of the populations fully cover the small range SR, i.e., there is at least one specimen reaching the lower and one reaching the upper limits of the SR, only 38% of the populations entail specimen with ray flower numbers over the entire broad range BR. The median over all populations is m = 46 with a standard deviation of sd = 8.3 and an overall interquartile range of IQR = 11. There is only one population (nb 23) whose IQR boundaries are not overlapping with the overall IQR showing that the distribution of ray flower numbers is quite stable mostly around SR with, however, slight variations of central values and function widths over all locations. The first location was sampled first at 3th April and the last location at 29^th^ May showing no temporal trend (p-value = 0.36) which indicates that there is no phenological bias in ray flower counts within the two-month spring period.

**Fig 4:**
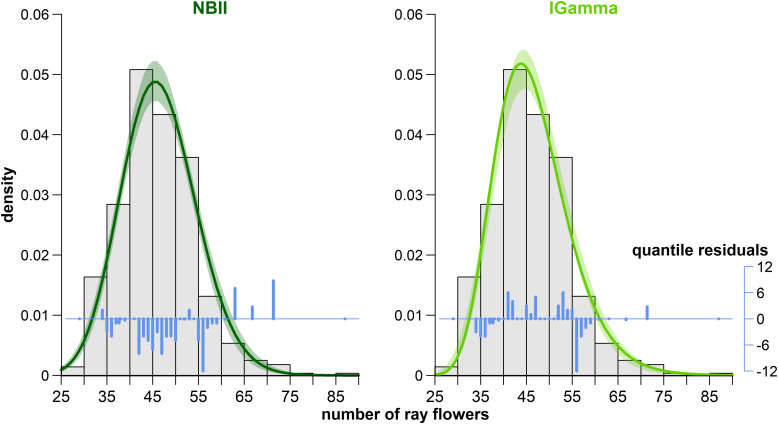
Probability density distributions on count data with bin size = 5 (grey bars) of n = 563 ray flower counts; modes are visible as positive or negative deviations from the theoretical probability density functions (discrete NBII: darkgreen and continuous IGamma: light green) through clusters of quantile residuals RQ (blue); uncertainty in the model fit was quantified using bootstrap resampling of the input counts, with shaded areas indicating 95% confidence intervals.

**Fig 5:**
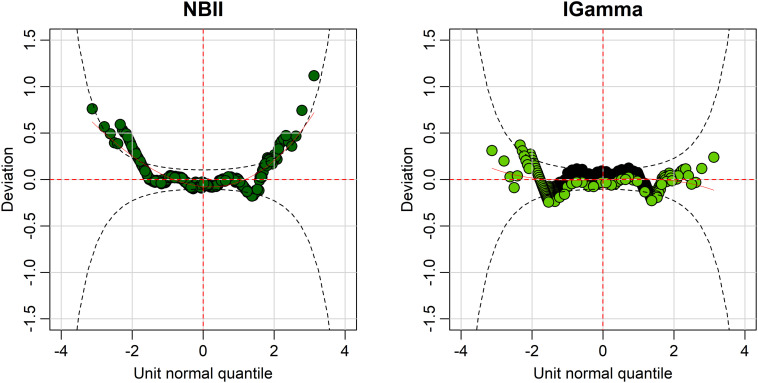
Worm plots comparing empirical and model-based quantiles for the negative binomial type II (NBII; dark green) and inverse gamma (IGamma; light green) fits, highlighting differences in tail behavior across the range of ray flower numbers.

Across all sampled locations, ray flower counts were best described by a negative binomial type II (NBII) distribution (AIC = 3956.9), reflecting pronounced overdispersion relative to a Poisson process (Fig 4, dark green curve showing the scaled NBII probability mass function). The maximum-likelihood estimate of the mean number of ray flowers was μ = 46.5 (95% CI: 45.8–47.2), with a dispersion parameter of σ = 0.45 (95% CI: 0.44–0.46). For frequency intervals greater than one, the NBII probability density function closely approximated the observed distribution of ray flower counts across individuals (see example with bins of size = 5 as grey bars in Fig 4). Model adequacy for the NBII distribution was assessed using randomized quantile residuals (RQRs). The residuals had a mean of 5.48 × 10^-5^ and a standard deviation of 0.992, demonstrating that the model is unbiased and properly scaled. The overdispersion index, calculated as the sum of squared residuals divided by the residual degrees of freedom, was 0.986, close to 1, confirming that the NBII model accurately captures the variance of the observed counts. The worm plot showed that most residuals fall close to the zero line within the central quantiles (−1.9 to 1.9), whereas extreme low and high counts resulted in larger residuals at the outer quantiles, reflecting the presence of rare but genuine tail observations (Fig 5 – NBII).

For comparison, a continuous Inverse Gamma (IGamma) distribution was also selected from fitted continuous distributions (AIC = 3937.04; Fig 4, green curve shown as the true probability density function). On the original scale, the estimated mean number of ray flowers was μ = 43.8 (95% CI: 42.7–44.9), with a dispersion parameter of σ = 0.173 (95% CI: 0.165–0.180). Randomized quantile residuals for the IGamma model showed a mean of 0.53, a standard deviation of 0.17, and an overdispersion index of 0.31, consistent with substantial underestimation of variance relative to the observed counts. In the corresponding worm plot, residuals remained close to zero across the distribution (Fig 5), including at the tails, which reflects that the IGamma model smooths extreme observations more strongly than the NBII model.

Overall, these diagnostics show that although the IGamma model produces visually tighter residuals, the NBII distribution provides a statistically more appropriate representation of discrete ray flower counts, capturing overdispersion and population-level variance despite some extreme observations appearing as tail outliers. Hence, although slight deviations from the fitted distribution models are expected, particularly at the tails, there is no apparent multimodal behavior that would be expected if, e.g., Fibonacci numbers would generate well pronounced individual distributions. In fact, modal morphologies can be made visible as outliers (O) exceeding the upper bounds of the 95% confidence interval (CI) obtained from the NBII distribution model, with O = {34, 42, 55, 72, 87}, and from the IGamma distribution, with O = {34, 55, 56}. Conversely, the values 38 and 44 fall below the lower 95% CI bound of the IGamma simulation.

Within the Broad Range (BR), the quantile residuals ${RQ}_{n}$ form different characteristic modes depending on the selected model. While NBII tends to generally underestimates empirical counts showing negative ${RQ}_{n}$ clusters at around 36, 46 and 56, the IGamma fit shows 5 modes that represent individual peaks at around 36, 42, 47, 53 and 56 (blue bars Fig 4). According to [1], there are two modes with negative residuals that mark a positive deviation from the theoretical distribution and three positive modes in the function center where ray flower counts occur less than it would be expected from the theoretical distribution

## Discussion

In this study, I demonstrate that the spatial and temporal distribution of ray flower numbers in *Bellis p.* follows a stochastic rather than deterministic pattern. Across different populations, no consistent numerical sequence controls ray flower formation, indicating the absence of discrete developmental constraints. Therefore, the statement that the number of ray flowers do have a conspicuous preference for Fibonacci numbers [27] is imprecise. In my study, the rank order of ray flower counts is 42 (x 37), 47 (x 33), 43 (x 30), 46 (x 29), **55** (x 27), 41 (x 27), 45 (x 26), **34** (x 25), 48 (x 23), 44 (x 23) and the following. The two Fibonacci numbers (marked as bold) are within a series of other frequent numbers. There is no prominent position recognizable that allows to highlight a certain number. In fact, most individuals exhibit ray flower numbers within the range of 40–48 across all sampled populations, providing strong evidence that ray flower number in *Bellis p.* can be regarded as a stochastic variable. Accordingly, the overall distribution of count data is adequately captured by a discrete negative binomial type II (NBII) model, with randomized quantile residuals (RQRs) closely approximating a standard normal distribution N(0,1), indicating a plausible stochastic fit to the data. Against this background, mechanisms of phyllotactic patterning based on numerical canalization during reiterative organogenesis, as proposed by, e.g., Battjes and Prusinkiewicz, 1998; Reinhardt et al., 2003; Zhang et al., 2021, may play only a subordinate role in the initiation of *Bellis p.* ray flower development, which was also reported for other Asteraceae species [43]. It rather supports the hypothesis that phyllotactic disorder is more frequent than predicted by classical models, arising as a consequence of stochastic noise in the genetic and molecular control of plant development, where fluctuations in auxin dynamics, cell growth, and signaling thresholds contribute to variability in primordium positioning (e.g., Bellows et al., 2023; Kitazawa, 2021; Refahi et al., 2016).

However, the graphical representation of the same ray flower counts reveals that at 34 and 55 a sharp peak is formed in each case (Fig 2). These peaks do not necessarily indicate high absolute numbers of counts (in comparison to the central region of the distribution), they rather show positive deviations from an expected stochastic distribution at respective positions and might therefore be indicated as outliers. Besides high counts at the extreme right tail (72 and 87), the NBII confidence-interval–based simulations identified outliers at 34, 42, and 55, whereas the IGamma simulation provides outliers at 34, 55, and 56. Both stochastic modelling frameworks consistently identified the numbers 34 and 55 as outliers. Notably, these values correspond to two Fibonacci numbers (34 and 55) and one composite value (42), representing the sum of the Fibonacci number 21. Such values have been reported previously in the literature as the ray flower numbers of *Bellis p.*[27], particularly for phyllotactic order in Asteraceae floral head development [46,47]. For example, spiral phyllotaxis in Asteraceae capitula frequently exhibits Fibonacci parastichy pairs such as 34 and 55, which arise as stable outcomes of geometric and dynamical models of primordium initiation and packing [43,46,48,49]. However, the present study provides little evidence for strong numerical determinism in *Bellis p.* ray flower number formation, as the Fibonacci numbers emerge only as local maxima within an otherwise stochastic distribution, characterized by a marked decline toward the tails (<34 and >55) and a comparatively gradual transition across intermediate values.

In a stochastic process, the formation of local maxima can be additionally identified plotting the quantile residuals ${RQ}_{n}$ for the theoretical best fit distribution using varying quantile sizes, in the present study quantiles between 2 and 20% using the NBII and IGamma distribution. It can be clearly shown that according to [1], negative values of ${RQ}_{n}$ indicate that at a particular quantile position, the sample quantiles are generally reached earlier, i.e., there is a positive deviation of *Bellis p.* ray flower counts while the magnitude of ${RQ}_{n}$ reveals how persistent this deviation is over all tested quantile sizes. Particularly, after the ray flower number 55 there is a strong tendency of positive deviation within different quantile sizes, although this effect can already be fully compensated representing ray flower counts as aggregated frequencies of bin size = 5 that adequately represent the theoretical distribution (Fig 4). However, using the discrete count modeling approach with a NBII distribution, positive deviations of the empirical count data can also occur within the central region of count values (negative ${RQ}_{n}$ cluster), indicating that the central area remains relatively variable across a broad range, which complicates the detection of outliers as local maxima. Only the Fibonacci numbers 34 and 55 represent relatively sharp and therefore robust deviations across both tested probabilistic models. Overall, both stochastic models provide an adequate framework for capturing the variability and distribution of ray flower counts in *Bellis p.*.

Stochastic processes can be described using probabilistic models, such as the discrete negative binomial type II model and the continuous inverse gamma probability density function, which may be useful for detecting what I refer to as “improbable recurrency”, revealing unusual deviations from expected stochastic patterns, such as modal morphologies in plant architecture. Improbability emerges from continuous probability distributions of, for example, natural traits. Their distribution models usually form predictive stochastic frameworks to capture variability and uncertainty in biological systems, such as phyllotactic disorder caused by intrinsic molecular and cellular noise [11,45]. In this context, numerical sequences appear as improbable exceptions forming isolated, discrete occurrences which become apparent only in relation to the underlying probabilistic structure, a perspective that will be important for future studies on Asteraceae species aiming to disentangle the gene regulatory networks controlling floral head architecture [50]. In this regard, improbable recurrency may be dismissed as a manifestation of a multimodal stochastic pattern around the expected regularities; it may rather indicate sharp accumulations of individuals sharing common developmental characteristics. Moreover, since recurrency can be mathematically constructed, the notion of improbable recurrency cannot simply be categorized as an anomaly or dismissed as an outlier, as is often the case with unusual deviations in data science [51,52]. In fact, recurrent patterns form individual modes at specific values, representing distinct attractors that seem to interrupt stochasticity through deterministic components. It is here that the pinpoints for the human mind are formed, enabling the recognition of deterministic patterns embedded in the stochastic dynamics of biological populations.

## Conclusion

The number of ray flowers in Common Daisy exhibits substantial variation that appears independent of both spatial factors (i.e., the sampled population locations) and temporal factors (i.e., the sampled phenological periods). Most individuals display ray flower counts between 34 and 56, with a small number of outliers on either end of the distribution. A clear modal morphology is evident, with a high frequency of individuals exhibiting ray flower numbers between 40 and 48, centered around a mode of 42 and a median of 46. Overall, the distribution of *Bellis p.* ray flower counts is well-approximated by stochastic model frameworks, specifically the discrete negative binomial type II model and the continuous inverse gamma probability density function. This indicates that there is no inherent tendency toward discrete, deterministic numerical sequences in the population-level distribution. However, in comparison to the stochastic distribution models, sharp local deviations are observed that are most stable over both distribution models at ray flower counts 34 and 55, two values that align with numbers in the Fibonacci sequence. These deviations do not necessarily reflect high absolute frequencies in comparison to the central region of the count distribution. Rather, they indicate that the quantiles of the distribution are reached earlier at these specific values, implying a relatively high density of individuals at these points that form local maxima. I refer to this statistical pattern as “improbable recurrency”, signifying that these Fibonacci-associated values are neither pure outliers in a stochastic sense nor components of a multimodal stochastic distribution. Instead, they represent genuinely deterministic components that only become apparent when viewed within the context of the overarching stochastic nature of population behavior. It demonstrates that deterministic structures can be emergent properties within fundamentally stochastic systems. This underscores the principle that stochasticity and determinism are not antagonistic, but co-constitutive dimensions of biological organization, and perhaps even facets of a shared quantitative logic through which the human mind seeks to describe reality.

## Supporting information

S1 File Raw count data for all locations W1-34. (CSV)

## References

[1] Baker A. Are there genuine mathematical explanations of physical phenomena? Mind. 2005;114:454.

[2] Brookfield JFY. Predictability and evolutionary determinism - the search for quantitative explanationquantitative explanation. Curr Biol. 2024;34(20):R960–4. doi: 10.1016/j.cub.2024.06.056 39437735

[3] Linsky B, Zalta EN. Naturalized platonism versus platonized naturalism. J Philos. 1995;92:525–55.

[4] Uzan J-P. Fundamental constants: from measurement to the universe, a window on gravitation and cosmology. 2024. doi: ArXiv241007281

[5] Adler I. A History of the study of phyllotaxis. Annals of Botany. 1997;80(3):231–44. doi: 10.1006/anbo.1997.0422

[6] Cox RT, Carlton C. Paleoclimatic influences in the evolution of periodical cicadas (Insecta: Homoptera: Cicadidae: Magicicada spp.). Am Midl Nat. 1988;:183–93.

[7] Lewontin RC, Cohen D. On population growth in a randomly varying environment. Proc Natl Acad Sci U S A. 1969;62(4):1056–60. doi: 10.1073/pnas.62.4.1056 5256406 PMC223613

[8] Yamagishi MEB, Shimabukuro AI. Nucleotide frequencies in human genome and fibonacci numbers. Bull Math Biol. 2008;70(3):643–53. doi: 10.1007/s11538-007-9261-6 17994268

[9] Dean AM, Shnerb NM. Stochasticity-induced stabilization in ecology and evolution: a new synthesis. Ecology. 2020;101(9):e03098. doi: 10.1002/ecy.3098 32443176

[10] Fujiwara M, Takada T. Environmental stochasticity. Els. 2017. 1–8.

[11] Kitazawa MS. Developmental stochasticity and variation in floral phyllotaxis. J Plant Res. 2021;134(3):403–16. doi: 10.1007/s10265-021-01283-7 33821352 PMC8106590

[12] Paldi A. Stochastic or deterministic? That is the question. Organizational Journal of Biological Sciences. 2020.

[13] Renard P, Alcolea A, Ginsbourger D. Stochastic versus deterministic approaches. Environ Model Find Simplicity Complex. 2013;:133–49.

[14] Vogel RM. Stochastic and deterministic world views. J Water Resour Plan Manag. 1999;125:311–3.

[15] Grigas A. The Fibonacci Sequence: Its history, significance, and manifestations in nature. 2013.

[16] Klar AJS. Plant mathematics: Fibonacci’s flowers. Nature. 2002;417(6889):595–595. doi: 10.1038/417595a 12050641

[17] Newell AC, Shipman PD. Plants and fibonacci. J Stat Phys. 2005;121:937–68.

[18] Omotehinwa TO, Ramon S. Fibonacci numbers and golden ratio in mathematics and science. Int J Comput Inf Technol. 2013;2:630–8.

[19] Cobb JN, Declerck G, Greenberg A, Clark R, McCouch S. Next-generation phenotyping: requirements and strategies for enhancing our understanding of genotype-phenotype relationships and its relevance to crop improvement. Theor Appl Genet. 2013;126(4):867–87. doi: 10.1007/s00122-013-2066-0 23471459 PMC3607725

[20] Pfennig DW. Phenotypic plasticity & evolution: causes, consequences, controversies. Taylor & Francis; 2021.

[21] Sommer RJ. Phenotypic plasticity: from theory and genetics to current and future challenges. Genetics. 2020;215(1):1–13. doi: 10.1534/genetics.120.303163 32371438 PMC7198268

[22] Sultan SE. Phenotypic plasticity for plant development, function and life history. Trends Plant Sci. 2000;5(12):537–42. doi: 10.1016/s1360-1385(00)01797-0 11120476

[23] Gurung V, Muñoz-Gómez S, Jones DS. Putting heads together: developmental genetics of the Asteraceae capitulum. Curr Opin Plant Biol. 2024;81:102589. doi: 10.1016/j.pbi.2024.102589 38955094

[24] Kitazawa MS, Fujimoto K. Relationship between the species-representative phenotype and intraspecific variation in Ranunculaceae floral organ and Asteraceae flower numbers. Ann Bot. 2016;117(5):925–35. doi: 10.1093/aob/mcw034 27052344 PMC4845808

[25] Zhang T, Elomaa P. Development and evolution of the Asteraceae capitulum. New Phytol. 2024;242(1):33–48. doi: 10.1111/nph.19590 38361269

[26] Gray A. Gray’s new manual of botany: a handbook of the flowering plants and ferns of the central and northeastern United States and adjacent Canada. American Book Company; 1908.

[27] Hegi G. Illustrierte Flora von Mitteleuropa: T. 3. Compositae I (Allgemeiner Teil). Berlin - Hamburg: Paul Parey. 1979.

[28] Karahan F. Morphology, anatomy, palynology and achene micromorphology of Bellis L. (Asteraceae) species from Turkey. Acta bot Croat (Online). 2020;79(1):59–67. doi: 10.37427/botcro-2020-006

[29] Siatka T, Kašparová M. Seasonal variation in total phenolic and flavonoid contents and DPPH scavenging activity of Bellis perennis L. flowers. Molecules. 2010;15(12):9450–61. doi: 10.3390/molecules15129450 21178900 PMC6259450

[30] Ciobanu LA. Bellis perennis-variations of physiological responses in urban conditions. Ann West Univ Timisoara Ser Biol. 2016;19:77.

[31] Sadeghi M, Ahmadi N, Keshtkar E. Evaluation of morphological and biochemical changes in bellis perennis under lead-contaminated soils. 2021.

[32] Kavalcioglu N, Acik L, Pinar NM. Comparative RAPD analysis and pollen structure studies of Bellis perennis L. Turk J Bot. 2010;34:479–84.

[33] Schmid B. Clonal growth in grassland perennials: III. Genetic variation and plasticity between and within populations of Bellis perennis and Prunella vulgaris. J Ecol. 1985;:819–30.

[34] Rigby RA, Stasinopoulos DM. Generalized Additive Models for Location, Scale and Shape. Journal of the Royal Statistical Society Series C: Applied Statistics. 2005;54(3):507–54. doi: 10.1111/j.1467-9876.2005.00510.x

[35] Stasinopoulos DM, Rigby RA. Generalized additive models for location scale and shape (GAMLSS) in R. J Stat Softw. 2008;23:1–46.

[36] Akaike H. Information Theory and an Extension of the Maximum Likelihood Principle. In: Second Int Symp Inf Theory, 1973.

[37] van Buuren S, Fredriks M. Worm plot: a simple diagnostic device for modelling growth reference curves. Stat Med. 2001;20(8):1259–77. doi: 10.1002/sim.746 11304741

[38] Dunn PK, Smyth GK. Randomized quantile residuals. J Comput Graph Stat. 1996;5:236–44.

[39] Stasinopoulos MD, Rigby RA, Heller GZ, Voudouris V, De Bastiani F. Flexible regression and smoothing: using GAMLSS in R. CRC Press, Taylor & Francis Group. 2017.

[40] Battjes J, Prusinkiewicz P. Modeling meristic characters of Asteracean flowerheads. Symmetry in plants. World Scientific. 1998. 281–312.

[41] Reinhardt D, Pesce E-R, Stieger P, Mandel T, Baltensperger K, Bennett M, et al. Regulation of phyllotaxis by polar auxin transport. Nature. 2003;426(6964):255–60. doi: 10.1038/nature02081 14628043

[42] Zhang T, Cieslak M, Owens A, Wang F, Broholm SK, Teeri TH, et al. Phyllotactic patterning of gerbera flower heads. Proc Natl Acad Sci U S A. 2021;118(13):e2016304118. doi: 10.1073/pnas.2016304118 33771923 PMC8020676

[43] Cooke TJ. Do Fibonacci numbers reveal the involvement of geometrical imperatives or biological interactions in phyllotaxis?. Bot J Linn Soc. 2006;150:3–24.

[44] Bellows S, Janes G, Avitabile D, King JR, Bishopp A, Farcot E. Fluctuations in auxin levels depend upon synchronicity of cell divisions in a one-dimensional model of auxin transport. PLoS Comput Biol. 2023;19(11):e1011646. doi: 10.1371/journal.pcbi.1011646 38032890 PMC10688697

[45] Refahi Y, Brunoud G, Farcot E, Jean-Marie A, Pulkkinen M, Vernoux T, et al. A stochastic multicellular model identifies biological watermarks from disorders in self-organized patterns of phyllotaxis. Elife. 2016;5:e14093. doi: 10.7554/eLife.14093 27380805 PMC4947393

[46] Douady S, Couder Y. Phyllotaxis as a dynamical self organizing process part I: the spiral modes resulting from time-periodic iterations. J Theor Biol. 1996;178:255–73.

[47] Prusinkiewicz P, Zhang T, Owens A, Cieslak M, Elomaa P. Phyllotaxis without symmetry: what can we learn from flower heads?. J Exp Bot. 2022;73(11):3319–29. doi: 10.1093/jxb/erac101 35275600 PMC9162182

[48] Lee HW, Levitov L. Universality in phyllotaxis: a mechanical theory. 2021.

[49] Swinton J, Ochu E. Novel Fibonacci and non-Fibonacci structure in the sunflower: results of a. Int J Plant Dev Biol. 2016;2:1–12. 10.1098/rsos.160091 PMC4892450 27293788

[50] Elomaa P, Zhao Y, Zhang T. Flower heads in Asteraceae-recruitment of conserved developmental regulators to control the flower-like inflorescence architecture. Hortic Res. 2018;5:36. doi: 10.1038/s41438-018-0056-8 29977572 PMC6026493

[51] Foorthuis R. On the nature and types of anomalies: a review of deviations in data. Int J Data Sci Anal. 2021;12(4):297–331. doi: 10.1007/s41060-021-00265-1 34368422 PMC8331998

[52] Rousseeuw PJ, Hubert M. Anomaly detection by robust statistics. WIREs Data Min & Knowl. 2017;8(2). doi: 10.1002/widm.1236

