# Supplementary material for: Variations in ray flower numbers of Common Daisy (Bellis perennis L.) – the hidden cues of the Fibonacci order
Source: PLoS One. 2026 Aug 17;21(8):e0348529. doi: 10.1371/journal.pone.0348529 (PMC13480633; doi:10.1371/journal.pone.0348529)
Supplement: Supplementary file 1 [file pone.0348529.s003.docx]

*I would like to thank the reviewer for the careful and critical engagement with my work. In particular, I am grateful for the time invested in thoroughly examining the study and for the valuable comments regarding both the statistical analysis and the philosophical implications of my conclusions. I have addressed all comments carefully, add model metrics i.e. error estimations, confidence intervals, overdispersion index etc., extended the statistical methods with another discrete count model fit and bootstrapped simulations for outlier detection.*

|  | **REVIEWER 2** | |
| --- | --- | --- |
| **#** | **Specific Comments:** | **Reply / Action** |
| 1. | “The sampling design in “Material and methods” is not fully specified for statistical independence and representativeness…” | Thanks for pointing to a more extensive explanation of sampling procedure. I need to clearly state here that within patches, individuals were selected using random point sampling. This approach does not account for fine-scale spatial clustering within patches but ensures unbiased individual selection, assuming that any internal spatial structure does not systematically influence the sampling process. I have clarified this at ll. 91-105 and also added some survey details ll. 86-87. |
| 2. | “The choice of an inverse gamma distribution for discrete count data is only loosely justified by the GAMLSS AIC comparison…” | Your comment here is highly valuable! Although continuous distributions (e.g. Inverse Gamma) show a good fit to my empirical data, particularly with regard to quantile-quantile mapping, it is common to describe integer counts with discrete distribution models. I do not want to break the standard statistic procedure and have now also implemented discrete count model fits. I also added basic model diagnostics to emphasize the difference between both model families. These changes affect method part ll. 106-131 and result part ll. 197-220 |
| 3. | “The definition and use of the quantile residual measure RQn in equation [1] require more rigorous justification. Summing differences between sample and theoretical quantiles over all quantile sizes from 2–20 % is an ad hoc construction, and the interpretation of negative RQn as “positive deviations” from the inverse gamma is not standard; without a formal test (e.g. bootstrap envelopes or simulation under the fitted model)…” | Your intervention here is highly appreciated. I am very thankful for that! First of all I have now implemented a bootstrap simulation of both distribution models and report outliers on the basis of the 95% confidence interval of simulated counts see method ll. 132-139 and result ll. 227-236 and discussion ll. 260-275. Second I want to keep my quantile-quantile comparison since this is an intuitive way to understand statistics particularly for the reader that is not famillar with qq-plots or simulated CI outlier estimates. The reason why it is intuitive is that it takes into account different quantile sizes and this way marks binning effects of complex data when statistically projected. Since in the formular the theoretical quantiles will be subtracted from the data quantiles, every time we get a negative shift at specific quantiles, means, that there are unusual high counts in the data because the “quantiles start earlier” i.e. are lower in the data. |
| 4. | “The interpretation of local peaks at 34 and 55 as evidence of “improbable recurrency” and deterministic Fibonacci-related structure is not backed by a quantitative assessment of how unlikely such peaks are under the fitted stochastic model. Given that counts at 34 and 55 are of similar magnitude to several neighboring values, a parametric bootstrap…” | “*Given that counts at 34 and 55 are of similar magnitude to several neighboring values…*” this statement is not supported by the data, in fact my argumentation is the opposite which I still state at different parts (see ll. 260-275.) because neighboring vales indeed “fall back” to the theoretical distribution. I have now introduced a bootstrap simulation for both NBII and IGamma distribution models which support my, indeed visually derived, hypothesis (see comment nb 3.). Thanks for pointing to the missing quantitative test! |
| 5. | “The conceptual claim that “improbable recurrency” represents deterministic components embedded in a fundamentally stochastic system currently rests on a single dataset from one species, one region, and one sampling season. Without replication across years, environments, or related Asteraceae species, it is difficult to know whether the observed pattern is robust, species specific, or even reproducible; this weakens the broader philosophical conclusions…” | With this statement you are probable right, however, here I wanted to give clear empirical evidence on the ray flower number of one species *Bellis perennis* that has been reported in many standard literatures wrongly. Me I am just a passionate scientist that will never get funded for bigger studies like that. My motivation is to present clean and straight scientific work with no ambitions for broad projects. Nevertheless, I will of course continue to collect more samples, different areas and other species to continuously collect empirical evidence and sharpen philosophical implications. I hope that for the moment also the content of a smaller study is worth publishing. |
| 6. | “The statement that the distribution “is best described” by the inverse gamma pdf would benefit from more conventional model diagnostics…” | Thanks for this hint. I have now added mean, dispersion parameter, with CI, also randomized quantile residuals mean and sd, overdispersion index and worm plots for the inspection of the selected continuous and discrete distribution models ll. 197-220 |
|  | *I would like to thank the reviewer for the critical remarks that helped to improve the quality of the study, particularly with regard to the statistical analyses.* | |
