# Supplementary material for: Variations in ray flower numbers of Common Daisy (Bellis perennis L.) – the hidden cues of the Fibonacci order
Source: PLoS One. 2026 Aug 17;21(8):e0348529. doi: 10.1371/journal.pone.0348529 (PMC13480633; doi:10.1371/journal.pone.0348529)
Supplement: Supplementary file 2 [file pone.0348529.s004.docx]

*I would like to thank the reviewer for the careful and critical engagement with my work. In particular, I am grateful for the time invested in thoroughly examining the study and for the valuable comments regarding the aspects of developmental biology, particularly giving strong review comments about plant developmental mechanisms and a crucial method hint for the consideration of only wild-type individuals.*

*I have carefully addressed all comments in an effort to improve the overall quality of the manuscript and to clarify the interpretation of numerical determinism. In this regard, the revised argumentation adheres strictly to the statistical evidence and avoids overinterpretation of specific numerical patterns.*

*Please see below my point by point responses:*

|  | **REVIEWER 3** | |
| --- | --- | --- |
| **#** | **Specific Comments:** | **Reply / Action** |
| 1. | “Absence of developmental context and recent literature  The manuscript treats Fibonacci numbers in Asteraceae flower heads primarily as a mathematical pattern to be verified or refuted statistically, but it does not engage with the substantial body of work on how such patterns arise developmentally. Particularly relevant is the study by Zhang et al. (2021, PNAS) on phyllotactic patterning in gerbera…” | Thanks for this comment. First of all, allow me to give a **general statement**:  Indeed, my intention was not to direct my study too much into phyllotaxis because I do not consider plant architecture itself, I am rather concentrating on the pure numbers without asking about the location/position of plant compartments (angles around the stem etc.). My introducing question is where can we find numbers in nature and how are they constituted (deterministic or stochastic). Thus, the pure number of ray flowers can crucially behave different than the actual arrangement of floral organs (see also Cooke 2006)  In the **previous reviews** I already added some reference with regard to phyllotactic patterns. See my response: *“However, you are right I should also refer to phyllotactic patterns since in general my study can also contribute to this field of science and therefore add some new literature citation in the introduction ll. 37 and discussion ll.298-302 part.*  *See: Adler, I., Barabe, D. and Jean, R.V., 1997. A history of the study of phyllotaxis. Annals of botany, 80(3), pp.231-244.”*  Finally, in the most recent version of my manuscript (after intensive statistical re-work), I actually state that “…**indicating the absence of discrete developmental constraints**.” ll. This is based on the clear statistical evidence that the number of ray flowers can be described by stochastic processes (significant distribution models). On the basis of my data, we must conclude that Bellis p. ray flower number is a stochastic variable, however, with two astonishing exceptions at 34 and 55 which are predictable and thus no true outliers.  Nevertheless, I highly appreciate the stronger perspective from developmental biology. This is why I included a new discussion part at ll. 265-273 and ll. 282-288 adding recent literature |
| 2. | “The curious case of 42 and 47: additive combinations of Fibonacci numbers  During the evaluation of this manuscript, it was noted that the most frequent ray floret counts in the sample are 42 (mode) and 47 (second-highest peak), which can be expressed as 34 + 8 and 34 + 13 respectively — both sums of Fibonacci numbers (1,1,2,3,5,8,13,21,34,55,89). While the authors do not comment on this, the pattern is striking and deserves attention in the Discussion….” | First of all, I do not know which version of my manuscript was read by the reviewer, because in the most recent version, that is a statistically highly adapted version, the number 47 cannot clearly be identified as statistical outlier. Here I did a bootstrapped 95% confidence interval to test on outliers of two stochastic distribution models getting O(NB11) = 34, 42, 55, 72, 87 and O(inverse gamma) = 34, 55, 56 ll. 234-238 and 278-280  Hence, although it might be obvious from the figure 2, the number 47 is not really an individual peak, it rather belongs to the function maxima described by NBII or inverse gamma distribution. In conclusion, from my data I cannot interpret the number 47 (second highest peak) as Fibonacci combination since it just might be an artefact of my sampling. In other words, the number 47 belongs to the stochastic distribution of ray flower numbers and does not mark an exceptional case considering statistical tests. This is different with the two numbers 34 and 55 that are always marked as numbers outside both(!) stochastic distribution functions. I have strongly emphasized this behavior to prevent overinterpretation see ll. 253-256, 288-291 |
| 3. | “Clarification of plant material: single vs. double-flowered forms  double-flowered morphs can have dramatically altered ray floret numbers due to homeotic conversion of disc florets, which would introduce a severe structural bias into the distribution. Although double-flowered Bellis is not available in the wild, still I request that the authors add a clear statement in the Methods indicating that only wild-type, single capitula were sampled, and that any obviously aberrant garden escapes were excluded.” | Thanks for that valuable hint. I did my best to visually prevent morph sampling, although I didn’t test it genetically. I include a statement based on your comment at ll. 100-102 “Only wild-type, single capitulum Bellis p. individuals with central yellow disc flowers were sampled; no obviously double-flowered morphs (e.g. horticultural garden escapes) were included” |
| 4. | “Terminology: The manuscript alternates between “ray flowers” and “ray florets.” Please use one term consistently.” | Thanks for pointing to that inconsistency, however in the most recent version of the manuscript (after the statistical review from reviewer 1 & 2) I couldn’t find any term “ray floret” except in the short explanation where I point to the fact that both terms exist as used in literature at ll. 74 |
| 5. | “Corrections: The abstract gives the mode as 42 and median as 46, but Figure 2 appears to show 42 and 47 as twin peaks. Confirm this data.” | You are right, in figure 2 you can see the mode of 42, however the median is a calculated statistical metric that is not necessarily a visual individual peak in the count plot. The median divides a distribution by the 50% quantile. So yes, I can confirm the correctness of the metrics. See also the result section ll. 188 that gives consistent numbers. You can also see that the mean of the discrete NBII distribution, that describes the data well, is μ = 46.5 ll. 202 |
|  | *I would like to thank the reviewer for the critical remarks that helped to improve the quality of the study, particularly with regard to the sampling and developmental constraints creating phyllotactic patterns.* | |
