# Supplementary material for: Variations in ray flower numbers of Common Daisy (Bellis perennis L.) – the hidden cues of the Fibonacci order
Source: PLoS One. 2026 Aug 17;21(8):e0348529. doi: 10.1371/journal.pone.0348529 (PMC13480633; doi:10.1371/journal.pone.0348529)
Supplement: Peer review history

# Peer review: Decision Letter 0

**DOI:** 10.1371/journal.pone.0348529.r001

**Authors:** Qinglin Meng

12 Dec 2025

Dear Dr. Neumann,

Thank you for submitting your manuscript to PLOS ONE. After careful consideration, we feel that it has merit but does not fully meet PLOS ONE’s publication criteria as it currently stands. Therefore, we invite you to submit a revised version of the manuscript that addresses the points raised during the review process.

This is a clearly written and engaging study that combines careful field sampling with an intriguing conceptual discussion of stochastic variation and Fibonacci-related structure in floral traits. For PLOS ONE’s standards, I would require more rigorous specification and justification of the statistical modeling choices (including consideration of discrete alternatives and formal tests or simulations for the local peaks at 34 and 55) and clearer documentation of the sampling design and independence assumptions; I recommend, in addition, making the full ray flower count data easily accessible in machine-readable form and more explicitly distinguishing between what is demonstrated by the data and what is proposed as a broader conceptual hypothesis.

Please submit your revised manuscript by Jan 26 2026 11:59PM. If you will need more time than this to complete your revisions, please reply to this message or contact the journal office at plosone@plos.org. When you're ready to submit your revision, log on to https://www.editorialmanager.com/pone/ and select the 'Submissions Needing Revision' folder to locate your manuscript file.

- A letter that responds to each point raised by the academic editor and reviewer(s). You should upload this letter as a separate file labeled 'Response to Reviewers'.
- A marked-up copy of your manuscript that highlights changes made to the original version. You should upload this as a separate file labeled 'Revised Manuscript with Track Changes'.
- An unmarked version of your revised paper without tracked changes. You should upload this as a separate file labeled 'Manuscript'.

If you would like to make changes to your financial disclosure, please include your updated statement in your cover letter. Guidelines for resubmitting your figure files are available below the reviewer comments at the end of this letter.

If applicable, we recommend that you deposit your laboratory protocols in protocols.io to enhance the reproducibility of your results. Protocols.io assigns your protocol its own identifier (DOI) so that it can be cited independently in the future. For instructions see: https://journals.plos.org/plosone/s/submission-guidelines#loc-laboratory-protocols. Additionally, PLOS ONE offers an option for publishing peer-reviewed Lab Protocol articles, which describe protocols hosted on protocols.io. Read more information on sharing protocols at https://plos.org/protocols?utm_medium=editorial-email&utm_source=authorletters&utm_campaign=protocols.

We look forward to receiving your revised manuscript.

Kind regards,

Qinglin Meng, Ph.D.

Academic Editor

PLOS One

Journal Requirements:

When submitting your revision, we need you to address these additional requirements.

1. Please ensure that your manuscript meets PLOS ONE's style requirements, including those for file naming. The PLOS ONE style templates can be found at

https://journals.plos.org/plosone/s/file?id=wjVg/PLOSOne_formatting_sample_main_body.pdf and

https://journals.plos.org/plosone/s/file?id=ba62/PLOSOne_formatting_sample_title_authors_affiliations.pdf

2. In your Methods section, please provide additional information regarding the permits you obtained for the work. Please ensure you have included the full name of the authority that approved the field site access and, if no permits were required, a brief statement explaining why.

3. We note that your Data Availability Statement is currently as follows: All relevant data are within the manuscript and its Supporting Information files.

Please confirm at this time whether or not your submission contains all raw data required to replicate the results of your study. Authors must share the “minimal data set” for their submission. PLOS defines the minimal data set to consist of the data required to replicate all study findings reported in the article, as well as related metadata and methods (https://journals.plos.org/plosone/s/data-availability#loc-minimal-data-set-definition).

For example, authors should submit the following data:

- The values behind the means, standard deviations and other measures reported;

- The values used to build graphs;

- The points extracted from images for analysis.

Authors do not need to submit their entire data set if only a portion of the data was used in the reported study.

If your submission does not contain these data, please either upload them as Supporting Information files or deposit them to a stable, public repository and provide us with the relevant URLs, DOIs, or accession numbers. For a list of recommended repositories, please see https://journals.plos.org/plosone/s/recommended-repositories.

If there are ethical or legal restrictions on sharing a de-identified data set, please explain them in detail (e.g., data contain potentially sensitive information, data are owned by a third-party organization, etc.) and who has imposed them (e.g., an ethics committee). Please also provide contact information for a data access committee, ethics committee, or other institutional body to which data requests may be sent. If data are owned by a third party, please indicate how others may request data access.

4. When completing the data availability statement of the submission form, you indicated that you will make your data available on acceptance. We strongly recommend all authors decide on a data sharing plan before acceptance, as the process can be lengthy and hold up publication timelines. Please note that, though access restrictions are acceptable now, your entire data will need to be made freely accessible if your manuscript is accepted for publication. This policy applies to all data except where public deposition would breach compliance with the protocol approved by your research ethics board. If you are unable to adhere to our open data policy, please kindly revise your statement to explain your reasoning and we will seek the editor's input on an exemption. Please be assured that, once you have provided your new statement, the assessment of your exemption will not hold up the peer review process.

5. We note that Figure 1 in your submission contain map images which may be copyrighted. All PLOS content is published under the Creative Commons Attribution License (CC BY 4.0), which means that the manuscript, images, and Supporting Information files will be freely available online, and any third party is permitted to access, download, copy, distribute, and use these materials in any way, even commercially, with proper attribution. For these reasons, we cannot publish previously copyrighted maps or satellite images created using proprietary data, such as Google software (Google Maps, Street View, and Earth). For more information, see our copyright guidelines: http://journals.plos.org/plosone/s/licenses-and-copyright.

We require you to either (a) present written permission from the copyright holder to publish these figures specifically under the CC BY 4.0 license, or (b) remove the figures from your submission:

a. You may seek permission from the original copyright holder of Figure 1 to publish the content specifically under the CC BY 4.0 license.

We recommend that you contact the original copyright holder with the Content Permission Form (http://journals.plos.org/plosone/s/file?id=7c09/content-permission-form.pdf) and the following text:

“I request permission for the open-access journal PLOS ONE to publish XXX under the Creative Commons Attribution License (CCAL) CC BY 4.0 (http://creativecommons.org/licenses/by/4.0/). Please be aware that this license allows unrestricted use and distribution, even commercially, by third parties. Please reply and provide explicit written permission to publish XXX under a CC BY license and complete the attached form.”

Please upload the completed Content Permission Form or other proof of granted permissions as an ""Other"" file with your submission.

In the figure caption of the copyrighted figure, please include the following text: “Reprinted from [ref] under a CC BY license, with permission from [name of publisher], original copyright [original copyright year].”

b. If you are unable to obtain permission from the original copyright holder to publish these figures under the CC BY 4.0 license or if the copyright holder’s requirements are incompatible with the CC BY 4.0 license, please either i) remove the figure or ii) supply a replacement figure that complies with the CC BY 4.0 license. Please check copyright information on all replacement figures and update the figure caption with source information. If applicable, please specify in the figure caption text when a figure is similar but not identical to the original image and is therefore for illustrative purposes only.

The following resources for replacing copyrighted map figures may be helpful:

USGS National Map Viewer (public domain): http://viewer.nationalmap.gov/viewer/

The Gateway to Astronaut Photography of Earth (public domain): http://eol.jsc.nasa.gov/sseop/clickmap/

Maps at the CIA (public domain): https://www.cia.gov/library/publications/the-world-factbook/index.html and https://www.cia.gov/library/publications/cia-maps-publications/index.html

NASA Earth Observatory (public domain): http://earthobservatory.nasa.gov/

Landsat: http://landsat.visibleearth.nasa.gov/

USGS EROS (Earth Resources Observatory and Science (EROS) Center) (public domain): http://eros.usgs.gov/#

Natural Earth (public domain): http://www.naturalearthdata.com/

6. Please remove your figures from within your manuscript file, leaving only the individual TIFF/EPS image files, uploaded separately. These will be automatically included in the reviewers’ PDF.

7. If the reviewer comments include a recommendation to cite specific previously published works, please review and evaluate these publications to determine whether they are relevant and should be cited. There is no requirement to cite these works unless the editor has indicated otherwise.

Additional Editor Comments:

This is a clearly written and engaging study that combines careful field sampling with an intriguing conceptual discussion of stochastic variation and Fibonacci-related structure in floral traits. For PLOS ONE’s standards, I would require more rigorous specification and justification of the statistical modeling choices (including consideration of discrete alternatives and formal tests or simulations for the local peaks at 34 and 55) and clearer documentation of the sampling design and independence assumptions; I recommend, in addition, making the full ray flower count data easily accessible in machine-readable form and more explicitly distinguishing between what is demonstrated by the data and what is proposed as a broader conceptual hypothesis.

[Note: HTML markup is below. Please do not edit.]

Reviewers' comments:

Reviewer's Responses to Questions

**Comments to the Author**

1. Is the manuscript technically sound, and do the data support the conclusions?

Reviewer #1: Partly

Reviewer #2: Partly

**********

2. Has the statistical analysis been performed appropriately and rigorously?

Reviewer #1: No

Reviewer #2: No

**********

3. Have the authors made all data underlying the findings in their manuscript fully available?

The PLOS Data policy

Reviewer #1: Yes

Reviewer #2: Yes

**********

4. Is the manuscript presented in an intelligible fashion and written in standard English?

Reviewer #1: Yes

Reviewer #2: Yes

**********

Reviewer #1:

This paper combines empirical sampling of Bellis perennis ray flower numbers in Brandenburg with parametric modeling and a new conceptual term (“improbable recurrency”) to discuss how Fibonacci-like regularities may emerge from stochastic trait distributions. The figures nicely summarize spatial sampling, raw counts, within-population variation, and the fitted distribution, but some methodological and interpretive aspects need clarification for a PLOS ONE audience.

1. The counting protocol for ray flowers, defined as plucking and counting ligules, is described briefly but does not address potential observer error or within-head counting variability. Given that the argument hinges on single-unit differences in counts (e.g. 33 vs 34 or 54 vs 55), some indication of repeatability (e.g. re-counting a subset of capitula by the same or a second observer) would be useful to show that small differences are biological rather than measurement noise.

2. The analysis of spatial and temporal patterns in the “Results” section reports that there is “no spatial trend or autocorrelation” and that sampling date shows “no temporal trend (p = 0.36),” but the underlying statistical tests are not described. Clarifying whether these statements are based on regressions of mean ray flower number vs coordinates/date, variograms, Moran’s I, or other spatial statistics would make the claims about spatial and phenological independence more transparent.

3. Figures 2–4 are central to the argument but could be more analytically informative. For example, Figure 2 shows raw counts per integer value without confidence intervals, Figure 3 uses box-plots per population without explicitly marking sample sizes, and Figure 4 overlays the inverse gamma curve without any indication of uncertainty around the fit; adding sample sizes, error bars, or simulated envelopes would help readers visually gauge how unusual the local peaks at 34 and 55 actually are.

4. The discussion downplays the possibility that the observed local maxima are simply the result of finite sampling from a unimodal distribution, emphasizing instead the philosophical implications of “improbable recurrency.” To better align with PLOS ONE’s empirical focus, the authors should distinguish clearly between what the data and model demonstrably show (a good inverse gamma fit with small local deviations) and what is a broader interpretive or philosophical extrapolation, which might be better framed as a hypothesis rather than a conclusion.

5. The introduction and discussion draw on a wide and interesting set of references in evolutionary biology, phenotypic plasticity, and stochastic vs deterministic modeling, but they devote relatively little space to existing quantitative work specifically on floral trait distributions in Asteraceae and related phyllotactic systems. Strengthening the link to prior empirical studies on intra-specific variation in capitulum traits and phyllotactic disorder would better situate this dataset within the existing literature and clarify which aspects of the present study are confirmatory versus novel.

Reviewer #2:

The manuscript analyzes ray flower numbers in Bellis perennis across 34 populations and fits an inverse gamma probability density function to describe the distribution, with local deviations at Fibonacci numbers 34 and 55 interpreted as “improbable recurrency.” The study aims to link stochastic variation in floral traits with underlying deterministic numerical patterns such as the Fibonacci sequence.

1. The sampling design in “Material and methods” is not fully specified for statistical independence and representativeness: individuals were sampled within 50–1000 m² per population, but the within-plot selection procedure, avoidance of clonal individuals, and potential spatial clustering are not described. This is important, because treating 563 individuals as independent draws from a single distribution without accounting for population-level structure may underestimate uncertainty and obscure hierarchical variability between populations.

2. The choice of an inverse gamma distribution for discrete count data is only loosely justified by the GAMLSS AIC comparison, and no alternative discrete distributions (e.g. Poisson, negative binomial, Poisson–lognormal, zero-truncated models) are reported. Since the subsequent detection of local deviations at 34 and 55 depends critically on the assumed continuous reference model, the authors should clarify which families were compared, provide parameter estimates with uncertainty, and explain why a continuous inverse gamma is preferable to discrete count models for ray flower numbers.

3. The definition and use of the quantile residual measure RQn in equation [1] require more rigorous justification. Summing differences between sample and theoretical quantiles over all quantile sizes from 2–20 % is an ad hoc construction, and the interpretation of negative RQn as “positive deviations” from the inverse gamma is not standard; without a formal test (e.g. bootstrap envelopes or simulation under the fitted model), it is hard to distinguish genuinely unusual modes from sampling noise.

4. The interpretation of local peaks at 34 and 55 as evidence of “improbable recurrency” and deterministic Fibonacci-related structure is not backed by a quantitative assessment of how unlikely such peaks are under the fitted stochastic model. Given that counts at 34 and 55 are of similar magnitude to several neighboring values, a parametric bootstrap or simulation study under the inverse gamma model to estimate the distribution of local maxima would be needed before concluding that these peaks cannot arise by chance.

5. The conceptual claim that “improbable recurrency” represents deterministic components embedded in a fundamentally stochastic system currently rests on a single dataset from one species, one region, and one sampling season. Without replication across years, environments, or related Asteraceae species, it is difficult to know whether the observed pattern is robust, species specific, or even reproducible; this weakens the broader philosophical conclusions drawn about the co-constitutive nature of stochasticity and determinism in biological organization.

6. The statement that the distribution “is best described” by the inverse gamma pdf would benefit from more conventional model diagnostics, such as residual plots, goodness-of-fit tests, and information about overdispersion. Presently, the reader sees only a fitted curve overlaid on binned data and the qualitative comment that “slight deviations” exist, which is insufficient to judge model adequacy, especially when those deviations are central to the article’s main argument.

**********

what does this mean?). If published, this will include your full peer review and any attached files.

If you choose “no”, your identity will remain anonymous but your review may still be made public.

**Do you want your identity to be public for this peer review?**  For information about this choice, including consent withdrawal, please see our Privacy Policy

Reviewer #1: No

Reviewer #2: No

**********

[NOTE: If reviewer comments were submitted as an attachment file, they will be attached to this email and accessible via the submission site. Please log into your account, locate the manuscript record, and check for the action link "View Attachments". If this link does not appear, there are no attachment files.]

To ensure your figures meet our technical requirements, please review our figure guidelines: https://journals.plos.org/plosone/s/figures

You may also use PLOS’s free figure tool, NAAS, to help you prepare publication quality figures: https://journals.plos.org/plosone/s/figures#loc-tools-for-figure-preparation.

NAAS will assess whether your figures meet our technical requirements by comparing each figure against our figure specifications.

# Author comment: Author response to Decision Letter 1

**DOI:** 10.1371/journal.pone.0348529.r002

27 Jan 2026

I am very grateful to the reviewers for their thorough and critical evaluation of my manuscript, which clearly highlighted the limitations of my empirical study, particularly regarding the distribution modeling, model metrics, and simulation tests. I have carefully addressed all comments from both reviewers and have revised the manuscript according to their suggestions.

In particular, I have added a second discrete count distribution model, employing a fitting routine analogous to that used for the IGamma approach. I have included goodness-of-fit metrics, confidence intervals, model residuals, and a new bootstrapped simulation for outlier detection. The Fibonacci analysis has now been conducted using both modeling approaches, incorporating the discrete standard model as well as the continuous IGamma approximation with discretization in the simulation procedure.

Additionally, I have clarified the sampling design, expanded the methodological descriptions, and included statistical metrics for temporal trends and spatial autocorrelation. Finally, I have refined the framing of my hypothesis to better align with the data and statistical context, and I have incorporated relevant literature on phyllotactic patterns.I ensure you that this work is original and has not been published or submitted for publication elsewhere. The presented material is from the author and the research is conducted with scientific integrity and known norms of science, whereas no duplications exist.

Attachment Submitted filename: Reviewer_2_Response.docx

# Peer review: Decision Letter 1

**DOI:** 10.1371/journal.pone.0348529.r003

**Authors:** Sarah Jose

3 May 2026

Dear Dr. Neumann,

Please submit your revised manuscript by Jun 15 2026 11:59PM. If you will need more time than this to complete your revisions, please reply to this message or contact the journal office at plosone@plos.org. When you're ready to submit your revision, log on to https://www.editorialmanager.com/pone/ and select the 'Submissions Needing Revision' folder to locate your manuscript file.

- A letter that responds to each point raised by the academic editor and reviewer(s). You should upload this letter as a separate file labeled 'Response to Reviewers'.
- A marked-up copy of your manuscript that highlights changes made to the original version. You should upload this as a separate file labeled 'Revised Manuscript with Track Changes'.
- An unmarked version of your revised paper without tracked changes. You should upload this as a separate file labeled 'Manuscript'.

If you would like to make changes to your financial disclosure, please include your updated statement in your cover letter. Guidelines for resubmitting your figure files are available below the reviewer comments at the end of this letter.

If applicable, we recommend that you deposit your laboratory protocols in protocols.io to enhance the reproducibility of your results. Protocols.io assigns your protocol its own identifier (DOI) so that it can be cited independently in the future. For instructions see: https://journals.plos.org/plosone/s/submission-guidelines#loc-laboratory-protocols. Additionally, PLOS ONE offers an option for publishing peer-reviewed Lab Protocol articles, which describe protocols hosted on protocols.io. Read more information on sharing protocols at https://plos.org/protocols?utm_medium=editorial-email&utm_source=authorletters&utm_campaign=protocols.

As the corresponding author, your ORCID iD is verified in the submission system and will appear in the published article. PLOS supports the use of ORCID, and we encourage all coauthors to register for an ORCID iD and use it as well. Please encourage your coauthors to verify their ORCID iD within the submission system before final acceptance, as unverified ORCID iDs will not appear in the published article. *Only*  the individual author can complete the verification step; PLOS staff *cannot*  verify ORCID iDs on behalf of authors.

We look forward to receiving your revised manuscript.

Kind regards,

Sarah Jose, Ph.D.

Staff Editor

PLOS One

Journal Requirements:

If the reviewer comments include a recommendation to cite specific previously published works, please review and evaluate these publications to determine whether they are relevant and should be cited. There is no requirement to cite these works unless the editor has indicated otherwise.

[Note: HTML markup is below. Please do not edit.]

Reviewers' comments:

Reviewer's Responses to Questions

**Comments to the Author**

Reviewer #1: All comments have been addressed

Reviewer #2: All comments have been addressed

Reviewer #3: (No Response)

**********

2. Is the manuscript technically sound, and do the data support the conclusions?

Reviewer #1: Yes

Reviewer #2: Yes

Reviewer #3: (No Response)

**********

3. Has the statistical analysis been performed appropriately and rigorously?

Reviewer #1: Yes

Reviewer #2: Yes

Reviewer #3: (No Response)

**********

4. Have the authors made all data underlying the findings in their manuscript fully available?

The PLOS Data policy

Reviewer #1: Yes

Reviewer #2: Yes

Reviewer #3: (No Response)

**********

5. Is the manuscript presented in an intelligible fashion and written in standard English?

Reviewer #1: Yes

Reviewer #2: Yes

Reviewer #3: (No Response)

**********

Reviewer #1: All the comments have been addressed perfectly; no further comments. Strongly recommend acceptance for publication.

Reviewer #2: Thank you for addressing the reviewer concerns thoroughly and improving the manuscript; the revision is clear, technically consistent, and ready for publication.

Reviewer #3: This study PONE-D-25-51179 presents an interesting empirical investigation into the distribution of ray floret numbers in Bellis perennis, and I appreciate the attempt to introduce the concept of “improbable recurrency” to characterize the interplay between stochastic variation and deterministic patterns. The dataset of 563 individuals from 34 populations provides a valuable basis for examining intraspecific variability. I wish to disclose that my own expertise lies primarily in the developmental biology of Asteraceae, and I do not consider myself qualified to provide a definitive assessment of the mathematical and statistical modeling aspects of this manuscript. I therefore recommend that the editor seek an additional review from a mathematician or statistician with experience in distribution modeling to assess the appropriateness and correctness of the quantitative analyses presented in this paper.

The manuscript addresses a genuinely interesting question and contains a valuable dataset. However, the discussion must be placed in the context of known developmental mechanisms. Major revision is recommended.

Here, I draw the authors' attention to several points raised during the evaluation process that could significantly strengthen the manuscript if adequately addressed.

1. Absence of developmental context and recent literature

The manuscript treats Fibonacci numbers in Asteraceae flower heads primarily as a mathematical pattern to be verified or refuted statistically, but it does not engage with the substantial body of work on how such patterns arise developmentally. Particularly relevant is the study by Zhang et al. (2021, PNAS) on phyllotactic patterning in gerbera, which demonstrates that during early head development, incipient bract primordia emerge in Fibonacci numbers through a mechanism of intercalary insertion on an expanding rim, without requiring a fixed golden divergence angle. This process of “numerical canalization” explains why certain Fibonacci numbers (e.g., 13, 21, 34, 55) appear as robust attractors in Asteraceae inflorescences.

I strongly recommend the authors discuss their findings in light of this developmental framework. The local frequency peaks at 34 and 55 that the authors identify as “improbable recurrencies” are precisely the numbers that would be predicted to be the most strongly canalized developmental outcomes. This connection would transform the paper from a purely statistical observation into a biologically grounded explanation, and it would considerably strengthen the argument that these peaks represent genuine deterministic signals rather than random fluctuations.

2. The curious case of 42 and 47: additive combinations of Fibonacci numbers

During the evaluation of this manuscript, it was noted that the most frequent ray floret counts in the sample are 42 (mode) and 47 (second-highest peak), which can be expressed as 34 + 8 and 34 + 13 respectively — both sums of Fibonacci numbers (1,1,2,3,5,8,13,21,34,55,89). While the authors do not comment on this, the pattern is striking and deserves attention in the Discussion. If the developmental ground state tends toward 34 (a Fibonacci number), the frequent appearance of 42 and 47 could reflect an additive module that builds upon this foundation during later stages of floret initiation. Conversely, such post-hoc numerical patterns can easily arise by chance, so the authors should discuss this observation with appropriate caution. I do not insist that the authors endorse this interpretation, but a brief, balanced discussion would enrich the paper.

3. Clarification of plant material: single vs. double-flowered forms

double-flowered morphs can have dramatically altered ray floret numbers due to homeotic conversion of disc florets, which would introduce a severe structural bias into the distribution. Although double-flowered Bellis is not available in the wild, still I request that the authors add a clear statement in the Methods indicating that only wild-type, single capitula were sampled, and that any obviously aberrant garden escapes were excluded.

4. Terminology: The manuscript alternates between “ray flowers” and “ray florets.” Please use one term consistently.

5. Corrections: The abstract gives the mode as 42 and median as 46, but Figure 2 appears to show 42 and 47 as twin peaks. Confirm this data.

**********

what does this mean?). If published, this will include your full peer review and any attached files.

If you choose “no”, your identity will remain anonymous but your review may still be made public.

**Do you want your identity to be public for this peer review?**  For information about this choice, including consent withdrawal, please see our Privacy Policy

Reviewer #1: No

Reviewer #2: No

Reviewer #3: No

**********

[NOTE: If reviewer comments were submitted as an attachment file, they will be attached to this email and accessible via the submission site. Please log into your account, locate the manuscript record, and check for the action link "View Attachments". If this link does not appear, there are no attachment files.]

To ensure your figures meet our technical requirements, please review our figure guidelines: https://journals.plos.org/plosone/s/figures

You may also use PLOS’s free figure tool, NAAS, to help you prepare publication quality figures: https://journals.plos.org/plosone/s/figures#loc-tools-for-figure-preparation.

NAAS will assess whether your figures meet our technical requirements by comparing each figure against our figure specifications.

# Author comment: Author response to Decision Letter 2

**DOI:** 10.1371/journal.pone.0348529.r004

31 May 2026

I would like to thank the reviewer for the careful and critical engagement with my work. In particular, I am grateful for the time invested in thoroughly examining the study and for the valuable comments regarding the aspects of developmental biology, particularly giving strong review comments about plant developmental mechanisms and a crucial method hint for the consideration of only wild-type individuals.

I have carefully addressed all comments in an effort to improve the overall quality of the manuscript and to clarify the interpretation of numerical determinism. In this regard, the revised argumentation adheres strictly to the statistical evidence and avoids overinterpretation of specific numerical patterns.

Attachment Submitted filename: Reviewer_3_Response.docx

# Editor report: Decision Letter 2

**DOI:** 10.1371/journal.pone.0348529.r005

**Authors:** Jan Rychtář

23 Jul 2026

Variations in ray flower numbers of Common Daisy (Bellis perennis L.) – the hidden cues of the Fibonacci order

PONE-D-25-51179R2

Dear Dr. Neumann,

We’re pleased to inform you that your manuscript has been judged scientifically suitable for publication and will be formally accepted for publication once it meets all outstanding technical requirements.

Within one week, you’ll receive an e-mail detailing the required amendments. When these have been addressed, you’ll receive a formal acceptance letter and your manuscript will be scheduled for publication.

An invoice will be generated when your article is formally accepted. Please note, if your institution has a publishing partnership with PLOS and your article meets the relevant criteria, all or part of your publication costs will be covered. Please make sure your user information is up-to-date by logging into Editorial Manager at Editorial Manager® and clicking the ‘Update My Information' link at the top of the page. For questions related to billing, please contact billing support.

If your institution or institutions have a press office, please notify them about your upcoming paper to help maximize its impact. If they’ll be preparing press materials, please inform our press team as soon as possible -- no later than 48 hours after receiving the formal acceptance. Your manuscript will remain under strict press embargo until 2 pm Eastern Time on the date of publication. For more information, please contact onepress@plos.org.

Kind regards,

Jan Rychtář

Academic Editor

PLOS One

Additional Editor Comments (optional):

All reviewer's comments have been addressed

Reviewers' comments:

# Editor report: Acceptance letter

**DOI:** 10.1371/journal.pone.0348529.r006

**Authors:** Jan Rychtář

PONE-D-25-51179R2

PLOS One

Dear Dr. Neumann,

I'm pleased to inform you that your manuscript has been deemed suitable for publication in PLOS One. Congratulations! Your manuscript is now being handed over to our production team.

At this stage, our production department will prepare your paper for publication. This includes ensuring the following:

* All references, tables, and figures are properly cited

* All relevant supporting information is included in the manuscript submission,

* There are no issues that prevent the paper from being properly typeset

You will receive further instructions from the production team, including instructions on how to review your proof when it is ready. Please keep in mind that we are working through a large volume of accepted articles, so please give us a few days to review your paper and let you know the next and final steps.

Lastly, if your institution or institutions have a press office, please let them know about your upcoming paper now to help maximize its impact. If they'll be preparing press materials, please inform our press team within the next 48 hours. Your manuscript will remain under strict press embargo until 2 pm Eastern Time on the date of publication. For more information, please contact onepress@plos.org.

You will receive an invoice from PLOS for your publication fee after your manuscript has reached the completed accept phase. If you receive an email requesting payment before acceptance or for any other service, this may be a phishing scheme. Learn how to identify phishing emails and protect your accounts at https://explore.plos.org/phishing.

If we can help with anything else, please email us at customercare@plos.org.

Thank you for submitting your work to PLOS ONE and supporting open access.

Kind regards,

PLOS ONE Editorial Office Staff

on behalf of

Dr. Jan Rychtář

Academic Editor

PLOS One
